# Gastrointestinal Parasites in Humans and Rhesus Macaques: A Cross‐Sectional Study in Bhaktapur, Nepal

**DOI:** 10.1002/hsr2.71568

**Published:** 2025-11-26

**Authors:** Sabina Chhetala, Roshan Babu Adhikari, Janak Raj Subedi, Tirth Raj Ghimire

**Affiliations:** ^1^ Central Department of Zoology, Institute of Science and Technology Tribhuvan University Kathmandu Nepal; ^2^ Alka Health Institute Pvt. Ltd. Lalitpur Nepal; ^3^ Nepalese Army Institute of Health Sciences (NAIHS) Kathmandu Nepal; ^4^ Third Pole Conservancy (TPC) Bhaktapur Nepal; ^5^ Animal Research Laboratory, Faculty of Science Nepal Academy of Science and Technology (NAST) Lalitpur Nepal; ^6^ Department of Zoology, Tri‐Chandra Multiple Campus Tribhuvan University Kathmandu Nepal

**Keywords:** ascaris, cryptosporidium, human, macaques, one health, zoonosis

## Abstract

**Background and Aims:**

Gastrointestinal parasitism in wild animals at the human‐wildlife interface may be a warning concern both for wildlife health and human health. The current study was conducted to determine the prevalence of gastrointestinal parasites in humans and rhesus macaques (*Macaca mulatta*) in the Nilbarahi area, an area with increasing human‐macaque interaction in Bhaktapur, Nepal.

**Methods:**

A total of 200 fecal samples (*N* = 200) were collected via a non‐invasive technique. Fecal samples of macaques (*N*_1_ = 100) were conveniently collected, while a purposive sampling was carried out for collecting human feces (*N*_2_ = 100). Microscopic examination of these samples was carried out via direct wet mount, concentration (flotation and sedimentation), and acid‐fast staining techniques.

**Results:**

The prevalence of intestinal parasites was 13% among humans and 81% among macaques. In humans, the identified parasites included protozoa, such as *Cryptosporidium* sp., nematodes, like *Ascaris lumbricoides*, and cestodes, such as *Taenia* sp. Moreover, the Chi‐square test indicated that females (20.37%) had a significantly higher prevalence rate than males (4.35%) (*p* < 0.05). Similarly, protozoa such as *Balantidium coli, Cryptosporidium* sp., *Cyclospora* sp., *Eimeria* sp., *Endolimax* sp., *Entamoeba* spp., *Giardia* sp., nematodes, like Ascarid, hookworms, Strongyles, and *Trichuris* sp., cestodes, such as Taeniid, and trematodes, like *Controrchis* sp. and *Fasciola* sp., were reported from macaques.

**Conclusion:**

The study reported a significantly greater prevalence and diversity of gastrointestinal parasites in macaques than in humans. Since a few species are implicated in zoonoses, the study highlights the need for a health awareness campaign and management of the macaque population in the study area. Additionally, public education, appropriate waste management near the temples, and controlled feeding to macaques must be implemented to reduce parasitism and spillover risks to humans.

## 1 Introduction

Gastrointestinal parasites (GIPs) reside in the intestinal tracts of hosts, including wild [1, 2] and domestic animals [3] and humans [4], and are the critical indicators of gastrointestinal (GI) health status. In clinical settings, the most common parasites include the single‐celled protozoa such as *Cryptosporidium parvum, Cyclospora cayetanensis, Entamoeba histolytica*, and *Giardia lamblia*, which are primarily associated with diarrheal symptoms [5]. In contrast, soil‐transmitted helminths (STHs), such as *Ascaris lumbricoides, Trichuris trichiura, Ancylostoma duodenale*, and *Necator americanus*, remained the most common helminths, disproportionately affecting the poorest and most disadvantaged ethnic communities worldwide [6, 7]. Additionally, other types of helminths, including cestoda: tapeworms, nematoda: pinworms and threadworms, and trematoda: flatworms, also frequently infect humans [8].

Nonhuman primates (NHPs) are the closest biological relatives of humans [9], and they offer valuable insights into human evolution, biology, and behavior [10]. Among them, rhesus macaques (*Macaca mulatta*, TSN: 180099, www.itis.gov) occur in broad geographical ranges with native populations across South, Central, and Southeast Asia and introduced populations in Florida, Puerto Rico, and China [11, 12] Concerning their parasitic richness, a wide range of intestinal protozoa (*Giardia lamblia, Balantidium coli, E. histolytica, Cryptosporidium* spp.) and helminths (*Schistosoma mansoni, Oesophagostomum* sp., *Enterobius vermicularis, Strongyloides* sp., *Trichostrongylus, Trichuris* sp., *Ascaris* sp., *Chabertia*, hookworm, and *Taenia* sp.) have been reported in them across different geographical landscapes globally [13, 14, 15]. These findings suggest that rhesus macaques could act as potential reservoirs of GIPs, which may have a deleterious health impact accompanied by severe morbidity and mortality.

However, it couldn't be ignored that most GIPs infections in the wild are often subclinical, and many species rely on tolerance strategies, implying that damage is minimized through tissue repair or compensating for resource loss and limiting immunopathology [16, 17] without harming the infecting parasites.

Notably, many GIPs infect both humans and macaques and possess zoonotic potentialities; therefore, the risks of parasitic cross‐transmission always remain higher at the human‐macaque interface [18, 19, 20]. For instance, human infection with *Strongyloides fuelleborni* in a community in Udon Thani province [21] and macaque‐borne *Plasmodium knowlesi*, a simian malaria [22, 23] in Southeast Asia, suggest direct zoonotic spillover from macaques to humans. In Nepal, this particular aspect of health threats remains more prominent in urban settings and religious sites, where a large population of macaque co‐exists with humans [14]. This indicates the necessity of research of this kind based on the One Health perspective linking human, domestic, and wild animals, for understanding transmission dynamics and designing effective control strategies.

To date, GIPs in humans and macaques have been studied separately in Nepal. For example, the literature [24, 25, 26] describes a wide range of GIPs in humans, and studies [14, 27, 28] have identified GIPs in macaques at different locations. However, the divergence and frequency of GIPs in these two host species living in the same geographical area have not been illustrated, and in‐depth knowledge about the species and number of parasites shared between these hosts and zoonotic risks is still lacking. Therefore, the current study aimed at a One‐health approach to survey parasites in both humans and macaques at the human‐macaque interface, around Nilbarahi temple areas in Bhaktapur, Nepal. Additionally, the study also seeks to identify probable risk factors for human parasitism by assessing biological factors (age, sex, behavior), environmental factors (sanitary practices, drinking water, defecation system, presence of animals nearby the household), and host factors, including deworming and health awareness.

## 2 Materials and Methods

### 2.1 Study Design

The study was a descriptive cross‐sectional study. The field study in the Bode of Madhyapur Thimi, Bhaktapur, Nepal, was conducted from May to June 2020.

### 2.2 Study Area

**Figure 1:**
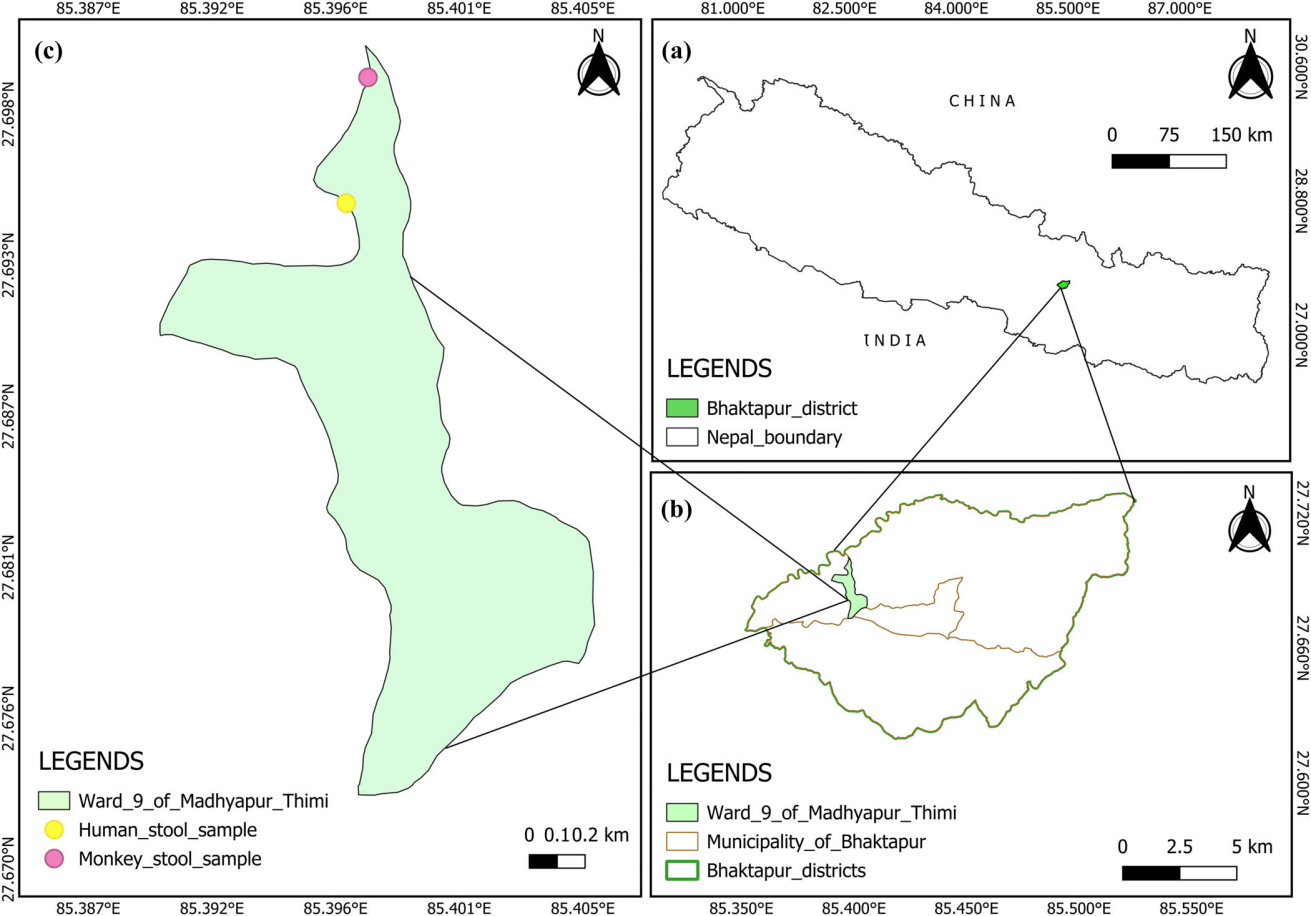
Map of the study area. (a) Map of Nepal. (b) Map of Bhaktapur district. (c) Map of Madhyapur Thimi Ward Number 9.

**Figure 2:**
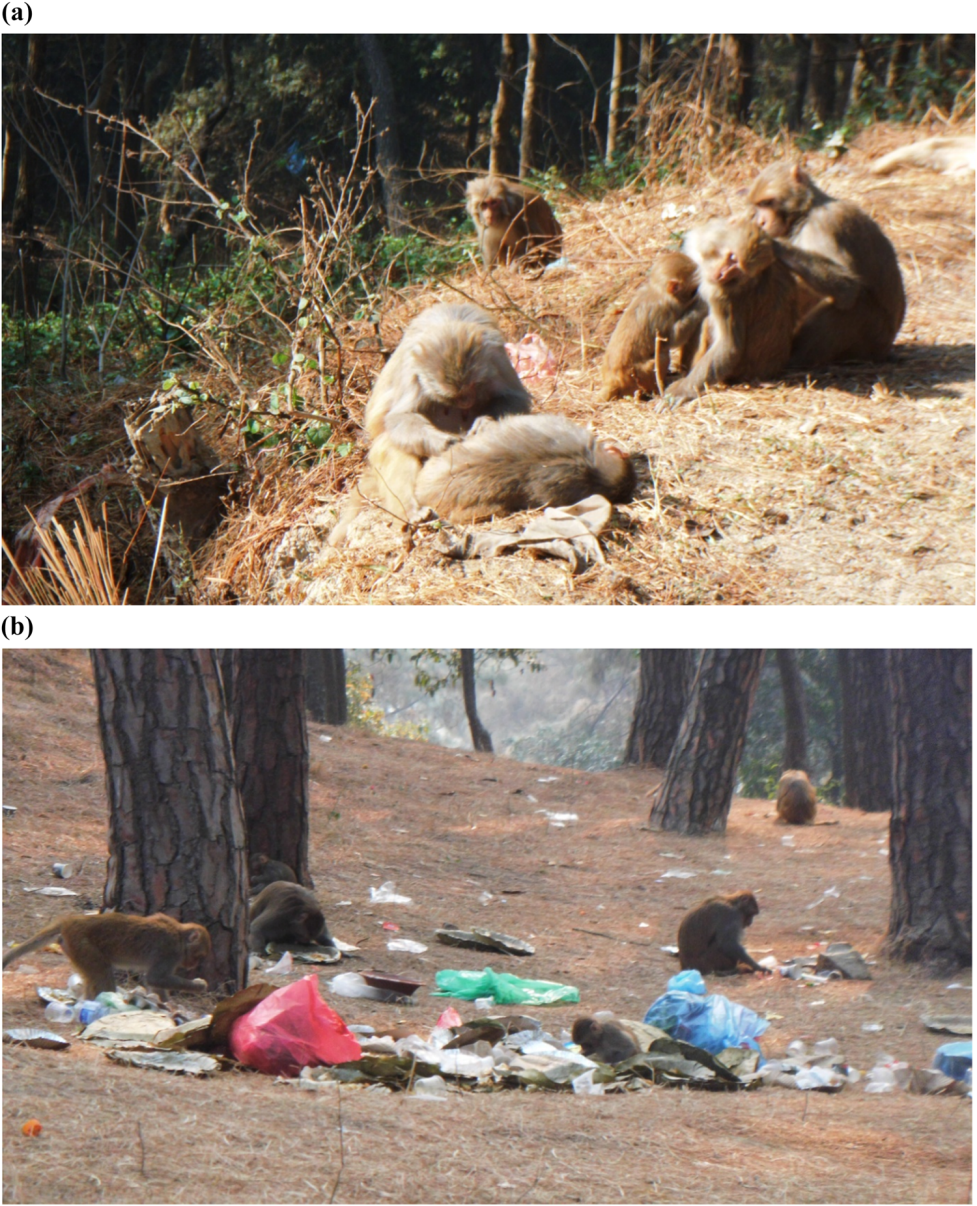
Rhesus macaques in the study area. (a) Grooming behavior. (b) Macaques raiding on garbage.

The study was conducted in the area around the Nilbarahi temple (27.695441, 85.396436, 1300 m asl), situated in the Bode of Madhyapur Thimi (Figure 1). Nilbarahi is one of the four Barahi temples in Kathmandu Valley and holds cultural and social significance for the surrounding communities. It is very often flooded with both domestic and international religious tourists and visitors. Notably, the temple is surrounded by a religious forest, which serves as the home for different wild animals, including the macaques. The estimated population of these macaques is 150 individuals, as assessed by direct counts and information from local people. These macaques frequently move into temple areas and nearby human settlements in search of food and drink, often foraging through garbage heaps (Figure 2). Therefore, due to the increasing macaque movements, the Nilbarahi area is epidemiologically significant for parasite transmission between humans and macaques and remains an ideal location for our study.

### 2.3 Ethical Consideration

This study was ethically approved by the Government of Nepal and the Nepal Health Research Council (NHRC) (Permission No.: 347/2021‐08‐25). The required permission for collecting the fecal samples was issued by the Ministry of Forests and Environment, Department of Forest and Land Management (Permission No.: 129/2077/078), Madhyapur Thimi Municipality, Bhaktapur (Permission No.: 789/2077/078), and Madhyapur Thimi Municipality Ward Number 9, Bhaktapur (Permission No.: 499/2077/078). Additionally, we obtained oral consent from local inhabitants to collect epidemiological data for evaluating potential risk factors.

### 2.4 Inclusion and Exclusion Criteria

The study included the Newar ethnic people aged 18 years and older who were able to talk regarding our objectives. But the people other than the Newar group, individuals under 18 years of age, who were unable to speak completely regarding our objectives, and individuals who did not want voluntary participation, were excluded from the study.

### 2.5 Human Sample Size Calculation

The formula for sample size estimation without population correction used was:

$n=\frac{z^{2}p\;(1–p)}{e^{2}}=45$, where estimated proportion (p) = 0.97 based on previous prevalence rates of 97.0% in ethnic people of Nepal [24], confidence level (Z) = 95%, margin of error (e) = 0.05, population size (*N*) = 3000. However, a total of 100 stool samples were collected to reduce margin of errors and make more strong data.

Therefore, a purposive sampling method was used to collect the fecal samples from 100 ethnic people (18 years or more), representing about 3.33% of the Bode area (*N* = 3000). The selection of those people was also based on the proximity to temple areas and the frequent macaque incursions into households from proximal forests.

### 2.6 Pilot Survey and Focal Group Discussion

A pilot survey with a pre‐structured questionnaire in a small group of ethnic people was conducted in April 2020. A questionnaire on the geographical landscape and surrounding environmental factors, including the forest type and estimated population size of macaques. We also conducted group discussions with local people (Health worker‐1, Farmer‐1, Housewife‐1, and Teacher‐1) for 1 hr to communicate the objectives of our study, questionnaire responses, and highlight the importance of this study. Information regarding macaques' visits to the temple and household areas was also noted. Finally, the questionnaire was tested, updated, validated, and finalized for the final field survey (Supporting Information S1).

### 2.7 Socio‐Demographic and Behavioral Data Collection

For the collection of human fecal samples, households were selected conveniently based on respondents' availability, acceptance of informed consent, and historical visits of macaques nearby them. Therefore, door‐to‐door visits and resident interactions were organized. The purpose of the study was explained to the participants, and informed consent was obtained from them. Then, an interview was taken with the participants (*N* = 100) with pre‐validated questionnaire that had the information on age, sex (biologically male or female), occupation, education levels, sources of drinking water, drinking habits, levels of environmental status, defecation habits, animal husbandry, knowledge on GI parasites, experienced symptoms (stomachache or diarrhea), consumed drug duration, visit frequency of macaques, and knowledge on macaque‐borne diseases in the areas.

### 2.8 Human Fecal Sample Collection and Transportation

First, during a door‐to‐door survey, each respondent was instructed to collect morning stool (about 10 gm) directly on the 20 mL plastic screw‐capped vials. The proper guidance for stool collection and the quantity of sample to be collected was provided to the respondents by the trained field researchers. Therefore, a total of 100 vials with fecal samples from 100 human participants and the corresponding questionnaires were assigned specific identification codes. Each fecal sample was examined for macroscopic parasites, such as adult stages and the properties of feces. The samples were then immediately preserved in a 2.5% potassium dichromate (K_2_Cr_2_O_7_) solution, which is considered the best preservative media for a long‐term storage and maintains the original morphology of protozoa cyst/oocyst and helminth egg without distortion [29], and transported to the Research Laboratory, where they were stored at 4°C in a refrigerator before laboratory investigation.

### 2.9 Assessment of Macaque Population

In May 2020, we conducted a weekly preliminary field survey to gather information concerning the geographical landscape and surrounding environmental factors, including the forest type and estimated population size of macaques. We also investigated the patterns of increasing human and macaque interactions alongside the density of the human population living near the temple, which served as the core study area for our current research. Therefore, with the discussion with the local people and forest authorities, the site of macaque troops was noted. Then, a count method was applied to determine the number of macaque populations in the nearby forests.

### 2.10 Macaque Fecal Sample Collection and Transportation

Following the initial survey, we aimed to collect both human and macaque fecal samples concurrently. To collect macaque fecal samples, we visited every possible defecation site, including temple peripheries, nearby households, roads, crop fields, forest edges, and picnic spots, every morning (6:30 a.m. to 8:30 a.m.). We only considered the fresh fecal samples and excluded the preexisting samples in the open environment. During every field survey, an individual macaque or its troop was followed until defecation was observed. Then, the freshly defecated samples were inspected with the naked eye for the fecal characteristics, such as color, consistency, and the presence of dead nematodes, detached gravid proglottids, mucus, and blood. Finally, with extreme care, we collected the samples into 20 mL sterile vials using a wooden applicator and gloved hands, avoiding contamination with soil, insects, or environmental exposure. These samples were also preserved in a 2.5% K_2_Cr_2_O_7_ solution and were transferred to the laboratory.

### 2.11 Laboratory Processing and Examination

Since each laboratory technique commonly employed in fecal analysis, including direct wet mount, sedimentation, and flotation techniques, has its unique strengths and limitations, we used all these techniques in combination to maximize detection sensitivity across parasite taxa [30, 31] in the current study. Additionally, samples microscopically positive for *Cryptosporidium*‐ and *Cyclospora*‐like oocysts were subjected to modified acid‐fast staining to obtain bright red colored‐oocysts [32, 33].

#### 2.11.1 Direct Wet Mount Technique

It was employed to detect the trophozoites, cysts, oocysts, and larval stages of the GIPs. A single drop of fecal samples preserved in 2.5% K_2_Cr_2_O_7_ solution was directly observed under a microscope at ×10 and ×40 with or without Gram iodine stain [34].

#### 2.11.2 Sedimentation Technique

For sedimentation purposes, approximately 2 gm of fecal sample preserved in a 2.5% K_2_Cr_2_O_7_ solution and 12 mL of 0.9% sodium chloride (NaCl) were crushed in a mortar and then passed through a metallic tea strainer into a 15 mL centrifuge tube [35]. Furthermore, the mixture was subjected to centrifugation at 1200 revolutions per minute (rpm) for 5 min. Finally, after discarding the supernatant, a single drop of the fecal sediment was placed on a glass slide containing two drops of Gram's iodine and examined under the microscope (×40) [35].

#### 2.11.3 Saturated Salt Flotation Technique

For the flotation method, the mixture of 12 mL of flotation media (45% NaCl) and the fecal sediment obtained after the sedimentation method was centrifuged (1200 rpm × 5 min), and without discarding the supernatant, the flotation media was added drop by drop to fill the tube [36]. Thereafter, a coverslip was placed at the mouth of the tube, touching the flotation media, and left undisturbed for 10 min. Finally, the coverslip was gently removed and placed on the glass slide for microscopic examination (×40) [37].

#### 2.11.4 Acid–Fast Staining Technique

It involves preparing thin smears using the fecal sediment obtained after initial centrifugation [33]. The smears were then fixed in absolute methanol (2 min), after complete air‐drying, and then flooded with the carbol fuchsin stain for about 15 min. Destaining was performed using acid alcohol, and the smears were counterstained with malachite green for a minute. Finally, the smears were rinsed with cool water and, after complete air‐drying, observed under a microscope (×100) using immersion oil [33].

### 2.12 Microscopic Photography, Parasite Measurement, and Identification

A light microscope (B‐383PLi, OPTIKA) was used to examine the fecal samples, and microscopic images of different parasitic stages were captured with an “SXView 2.2.0.172 Beta” camera attached to the microscope. ImageJ (National Institute of Health) software version (64‐bit) was employed to measure the size of the detected parasitic stages. The parasite identification was confirmed based on morphological characteristics, such as shape, size, and staining properties of parasitic cysts, oocysts, ova, and larvae. Parasites were identified up to the generic level following the taxonomic details explained in the literature [14, 38, 39, 40, 41, 42, 43]. Furthermore, we distinguished hookworm and strongyle‐type ova by their morphological differences. Smaller ova, about 55–75 µm × 36–40 µm, with a thin, smooth shell and segmented blastomeres that often occupy most of the egg space, were classified as hookworm [44]. Larger ova, approximately 70–120 µm × 40–80 µm, more elongated with segmented blastomeres, and occupying less of the egg space during fresh fecal examination, were classified as strongyles [38]. Additionally, our methodology does not include culturing faecal samples until L3 larvae are obtained, which could enable species‐level identification of larger‐sized eggs [45], so we preferred to call them Strongyle in the current study.

### 2.13 Data Analysis

The collected data were encrypted and entered into a Microsoft Excel 2013 spreadsheet. Statistical analysis was performed via GraphPad Prism 5.00 (2007). The percentage prevalence of each reported parasite was calculated by dividing the total number of positive cases (infected hosts) by the total number of sampling population (hosts examined) and finally multiplying by 100 [45].

Prevalence(%)=totalnumberofhostsexamined/numberofinfectedhosts×100.

Diversity (Species richness) denotes the variety of species present in all the fecal samples. Similarly, concurrence represents the species present either in individual species or mixed species with others in those samples. The differences in diversity, prevalence, and concurrence of each parasite species were analyzed statistically using two‐sided Fisher's exact tests for bivariate or Chi‐square by trends tests for multivariate. Similarly, risks were analyzed using relative risk (RR), odds ratio (OR), and likelihood ratio (LR) with 95% confidence intervals (CIs) [46]. The RR denotes the risk ratio of parasitosis in the exposed versus the unexposed group. The OR represents the odds ratio of parasitosis in the exposed versus the unexposed group. Similarly, the LR indicates how much a diagnostic test result will change the odds of having parasitosis [46].

## 3 Results

### 3.1 Prevalence and Diversity of GIPs in Humans and Macaques

**Figure 3:**
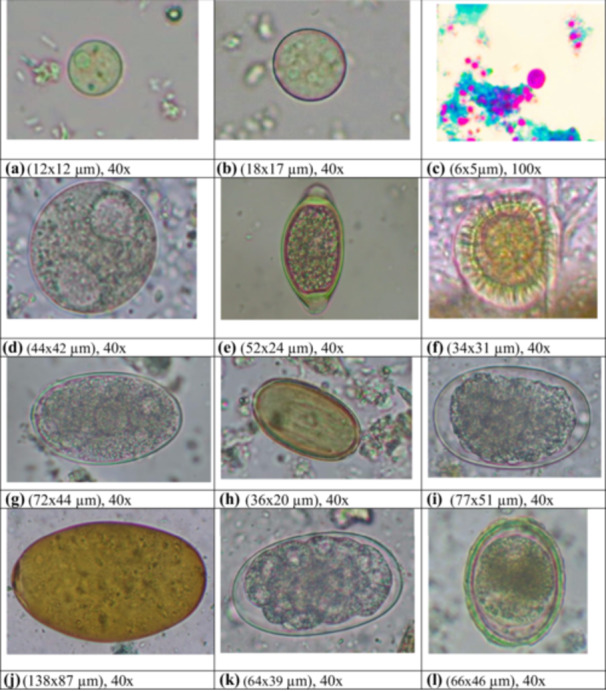
Parasitic cyst, oocyst, and ova under a compound microscope. (a) Cyst of *Entamoeba* sp., (b) Cyst of *Entamoeba coli*, (c) Oocyst of *Cryptosporidium* sp., (d) Cyst of *Balantidium coli*, (e) Egg of *Trichuris* sp., (f) Egg of Taenid, (g) Egg of Strongyle 1, (h) Egg of *Controrchis* sp., (i) Egg of Strongyle 2, (j) Egg of *Fasciola* sp., (k) Egg of hookworm, (l) Egg of *Ascaris* sp.

In this study, the prevalence rate of GIPs in the 100 fecal samples of humans was 13% (13/100). Considering the diversity, three different GIPs (one protozoan, one cestode, and one nematode) were reported. Additionally, most individuals exhibited single‐species parasitism (Table 1 and Figure 3).

**Table 1:** Prevalence, diversity, and concurrence of GIPs in humans and rhesus macaques in Bhaktapur, Nepal.; RR: Relative Risk, OR: Odds Ratio, LR: Likelihood Ratio, while comparing the GIP species between macaques and humans. The *p*‐values indicate the probability obtained from the Fisher's exact test (two‐sided).

Parasites	Macaques (*N* = 100)	Humans (*N* = 100)	*p*‐values	RR	OR	LR
Protozoa	76 (76%)	7 (7%)	< 0.0001	10.86 (5.269–22.37)	42.07 (17.19–103.0)	4.464
Sarcodina						
*Entamoeba* spp.	83 (83%)	0 (0%)	—	—	—	—
*Endolimax* sp.	1 (1%)	0 (0%)	—	—	—	—
Mastigophora						
*Giardia* sp.	4 (4%)	0 (0%)	—	—	—	—
Ciliata						
*Balantidium coli*	31 (31%)	0 (0%)	—	—	—	—
Apicomplexa						
*Cryptosporidium* sp.	18 (18%)	7 (7%)	< 0.05	2.571 (1.123–5.886)	2.916 (1.159–7.335)	1.537
*Cyclospora* sp.	5 (5%)	0 (0%)	—	—	—	—
*Eimeria* sp.	3 (3%)	0 (0%)	—	—	—	—
Helminth	24 (24%)	6 (6%)	< 0.005	4.000 (1.708–9.366)	4.947 (1.924–12.72)	1.789
Trematoda						
*Fasciola* sp.	1 (1%)	0 (0%)	—	—	—	—
*Controrchis* sp.	1 (1%)	0 (0%)	—	—	—	—
Cestoda						
Taeniid	3 (3%)	2 (2%)	> 0.99	1.500 (0.2560–8.789)	1.515 (0.2476–9.274)	1.206
Nematoda						
Ascarid	10 (10%)	5 (5%)	< 0.99	2.000 (0.7088–5.644)	2.111 (0.6945–6.418)	1.370
Strongyle	10 (10%)	0 (0%)	—	—	—	—
Hookworm	2 (2%)	0 (0%)	—	—	—	—
*Trichuris* sp.	1 (1%)	0 (0%)	—	—	—	—
Concomitance						
Single infection	29 (29%)	12 (92.3%)	< 0.005	2.417 (1.309–4.462)	2.995 (1.426–6.291)	1.584
Double infection	26 (26%)	1 (7.7%)	< 0.0001	26.00 (3.595–188.0)	34.78 (4.612–262.3)	2.251
Triple infection	15 (15%)	0 (0%)	—	—	—	—
Quadruple	11 (11%)	0 (0%)	—	—	—	—

In contrast, the prevalence of GIPs in macaques was 81% (81/100), and 14 diverse GIPs (seven protozoa, two trematodes, one cestode, and four nematodes) were reported. In macaques, the concomitant pattern of infections is reported to be higher than monoparasitism, and interestingly, concomitant infections up to four concurrent parasite species were detected (Table 1 and Figure 3).

Fisher's exact tests revealed significantly different prevalence rates of total protozoa (*p* < 0.0001) and total helminths (*p* < 0.005) and doubled infection rates (*p* < 0.0001) in macaques compared with those in humans. However, the frequency of single‐species parasitism in humans was significantly higher than in macaques (*p* < 0.005). Strong results in the context of statistical significance were obtained when the patterns of RR and OR of probability of specific parasite or groups of parasites occurring in humans and macaques were compared with 95% CIs. For example, the odds of protozoan infection were 42.07 times higher (95% CI: 17.19–103.0), those of helminth infection were 4.947 times higher (95% CI: 1.924–12.72) and those of double infection were 34.78 times higher (95% CI: 4.612–262.3) in macaques (Table 1).

### 3.2 Risk Factor Analysis for GIPs in Humans

In the next step, risk factor analysis indicated that female participants had a higher prevalence rate of GIPs than had male participants (20.37% vs. 4.35%), with a statistically significant difference (*p* < 0.05). In addition, males were infected with only *Ascaris lumbricoides* and *Taenia* sp. out of the three diverse species. However, analysis by RR, OR, and LR also did not reveal a strong association between parasites and gender. Employment status, such as being either unemployed or employed in farming, business, or labor, was positively associated with parasites, and the data were also statistically significant (*p* < 0.005). Interestingly, all three reported species were found in farmers, whereas *Cryptosporidium* sp. was present in businessmen, and *Ascaris lumbricoides* was present in laborers. However, the prevalence rates of GIPs did not significantly differ across various age groups, although the rates increased by more than two times from the 18–40‐year‐old age group to the more than 60‐year‐old age group. Furthermore, education level, sanitary conditions of homes, defecation systems, sources of drinking water, water consumption methods, the presence of nearby pets and macaques, knowledge of zoonotic diseases related to macaques, and history of deworming did not significantly influence GIP infection (*p* > 0.05) (Table 2).

**Table 2:** Risk factors of gastrointestinal parasitic infection in humans in Bhaktapur, Nepal. The *p*‐values were calculated using two‐tailed Fisher's (F) exact tests or Chi‐square by trends. DF: degree of freedom.

Variables of risk factors	Sub variables	Respondents *N* = 100	Positive *n* (%)	*p*‐values	RR, OR, LR
Gender	Male	46	2 (4.35)	< 0.05, F	RR: 0.2134, 95% CI: 0.04983–0.9143; OR: 0.1777, 95% CI: 0.03717–0.8494; LR: 0.3042
	Female	54	11 (20.37)		
Age‐groups	18–40 yrs	58	5 (8.62)	< 0.5, (DF = 2)	
	41–60 yrs	33	6 (18.18)		
	Above 60 yrs	9	2 (22.22)		
Education	Primary	41	4 (9.76)	< 0.1 (DF = 3)	
	Secondary	8	0 (0.00)		
	Higher studies	20	1 (5.00)		
	Uneducated	31	8 (25.81)		
Employment	Business	17	2 (11.76)	< 0.005 (DF = 5)	
	Student	26	0 (0.00)		
	Labor	20	1 (5.00)		
	Teacher	3	0 (0.00)		
	Farmer	10	2 (20.00)		
	Unemployment	24	8 (33.33)		
Parasite knowledge	Yes	72	8 (11.11)	< 0.99, F	RR: 0.6222, 95% CI: 0.2224–1.741; OR: 0.5750, 95% CI: 0.1706–1.938; LR: 0.8365
	No	28	5 (17.86)		
Sanitary system	Good	81	8 (9.88)	< 0.1	RR: 0.3753, 95% CI: 0.1381–1.020; OR: 0.3068, 95% CI: 0.08743–1.077; LR: 0.7334
	Not perfect (based on nearby latrine, animals, and their dung)	19	5 (26.32)		
Toilet system	Pour flush latrine	89	10 (11.24)	< 0.5 (DF = 2)	
	Pit latrine	8	3 (37.50)		
	Open defecation	3	0 (0.00)		
Drinking water sources	Tap	97	12 (12.37)	< 0.5, F	RR: 0.3711, 95% CI: 0.06875–2.004; OR: 0.2824, 95% CI: 0.02374–3.358; LR: 0.9448
	Tap & Jar	3	1 (33.33)		
Way of drinking water	Direct use	35	7 (20.00)	< 0.5, (DF = 4)	
	Filter	38	5 (13.16)		
	Filter, boiling	24	0 (0.00)		
	Boiling	2	1 (50.00)		
	Direct use, filter	1	0 (0.00)		
Closest animal to home	Dog	46	7	< 0.99 (DF = 8)	
	Dog, monkey	36	4		
	Dog, monkey, cow	1	0 (0.00)		
	Dog, monkey, hen	5	0 (0.00)		
	Dog, hen	1	0 (0.00)		
	Dog, hen, cow	1	0 (0.00)		
	Goat	1	0 (0.00)		
	Hen	8	2 (25.00)		
	Monkey	1	0 (0.00)		
Monkey frequency near home	> Twice in a month	72	9 (12.50)	< 0.99, F	RR: 0.8750, 95% CI: 0.2930–2.613; OR: 0.8571; 95% CI: 0.2411–3.048; LR: 0.9560
	Once in a month	28	4 (14.29)		
Monkey defecate near home	Yes	71	9 (12.68)	< 1.5, F	RR: 0.9190, 95% CI: 0.3071–2.750; OR: 0.9073, 95% CI: 0.2557–3.219; LR: 0.9715
	No	29	4 (13.79)		
Knowledge of zoonosis by monkeys	Yes	4	1 (25.00)	< 0.5, F	RR: 2.000, 95% CI: 0.3378–11.84; OR: 2.333, 95% CI: 0.2240–24.30; LR: 2.231
	No	96	12 (12.50)		
Dewormed duration (before)	1 month	2	0 (0.00)	< 0.99 (DF = 3)	
	4–6 months	2	1 (50.00)		
	7–12 months	3	0 (0.00)		
	> 12 months	93	12 (12.90)		

### 3.3 Common GIPs in Humans and Macaques

Interestingly, we also compared the detailed morphological characteristics of the GIPs, which were common in both human and macaque hosts. Photomicrography revealed that the oocyst morphology of *Cryptosporidium* sp. was similar in both of these hosts, while the taeniid egg in humans was slightly greater than reported from macaques. Additionally, two morphological forms of Ascarid eggs were reported in macaques: one egg type was similar to the corticated egg of *A. lumbricoides*, while another was similar to *Toxocara* eggs, which were not present in humans (Supporting Information S2). Similarly, based on the size and characteristics of the cysts, four different morphological forms of *Entamoeba* were also detected in the macaque population (Supporting Information S3).

## 4 Discussion

The current study assessed the prevalence rates and diversity of GIPs in humans and macaques. The current prevalence rate in humans was lower (13%, *n* = 100) than that reported in previous studies conducted in central (97%, *n* = 100) and eastern (81%, *n* = 200) Nepal [24, 25]. The current rate is also slightly lower than the 16.84% prevalence reported among schoolchildren in the same district (*n* = 190) [26]. The differences in the prevalence rates of parasites across these studies might be attributed to the variation in three factors contributing to the disease triangle: primarily host factors, (including age, sex, immune status, and behavioral practices), the surrounding environment, and the nature of infecting parasites [47]. The current study recorded only three taxa, *Cryptosporidium* sp., *Ascaris lumbricoides*, and *Taenia* sp., although previous studies in school‐going children reported eight GIPs from the same district [26]. Considering the concurrency of parasitic infection, similar to the findings of earlier studies, the current data showed that the single infections were greater than those of concomitant infections among the participants. The low prevalence and low diversity of GIPs in the currently studied populations might be due to the distribution of routine anthelminthic drugs (e.g., albendazole) to Nepalese children [48]. For example, millions of doses of albendazole have been distributed to school‐going children nationwide, which has previously been shown to reduce the prevalence of STHs infections (from 74% to 51%), the proportion of heavy‐intensity infections (from 9% to 2%), and anemia (from 47% to 11%) [49, 50, 51]; recently, the successful role of albendazole in reducing helminth infection in the Ethiopian population has been reviewed [52, 53]. Despite the participants in the current study being adults (18–60+ years), mass drug administration benefited indirectly by decreasing environmental contamination and reducing parasite circulation in the study area, thereby influencing adult infection rates. Additionally, behavioral factors such as increased awareness of personal hygiene and access to clean water among adults may have played a role.

In contrast to the findings of GIPs in humans, a higher prevalence rate and more diverse GIPs in the macaque population were reported. The prevalence rates of GIPs ranged from 39.2% to 100% in macaques in different landscapes of Nepal [14, 27, 28, 54]. In Bangladesh, 100% of macaques were identified with at least a GIP [55], whereas across various landscapes in India, the prevalence ranged from 40% to 66.5% [56, 57]. These differences in GI parasite prevalence among macaques in Nepal, India, and Bangladesh, as reported in these studies, likely reflect variations in ecological, behavioral, and anthropogenic factors influencing parasitic exposure, which, when combined, might rule out differences in parasitic prevalence in the wild.

The present study identified 14 species of GIPs in macaques, along with four morphologically different cysts of *Entamoeba* with different fecal characteristics, indicating that macaques are critical sources or reservoirs of amebas with different pathogenic statuses in Nepal. The predominance of *Entamoeba* spp. in the present study was on par with the findings of earlier studies in Nepal [14] and China [58]. The higher prevalence observed in this study may be due to natural infections in macaques [59, 60] and the relatively simple and monogenetic life cycle pattern of amebas [61]. Previous research has revealed the presence of *E. histolytica, E. nuttalli, E. dispar, E. moshkovskii, E. hartmanni, E. chattoni*, and *E. polecki* in rhesus macaques worldwide [59, 62], suggesting that further molecular studies of *Entamoeba* species may indicate potential cross‐species transmission.

Among the helminths, Ascarid and Strongyle were the most commonly identified parasites in the current macaque population. This finding aligns with a study conducted in a temple‐centered community forest area of the neighboring district, Lalitpur, Nepal [14]. The acquisition of these STHs may be attributed to macaques' behaviors and habits, which involve significant exposure to the ground while searching for food and playing, particularly in areas around temples and households. Additionally, other usual behaviors such as frequent self‐grooming and anal region inspection may also contribute to the transmission of certain parasites. While grooming helps remove ectoparasites [63], which is beneficial, it can also inadvertently promote the spread of internal parasites due to increased social interactions [64]. For example, intestinal parasites such as *Trichuris trichiura* and ascarid worms have been found in fecal samples, with variations in infection rates linked to social rank and the intensity of behaviors [64]. Similarly, a previous research on Tibetan macaques (*Macaca tibetana*) has also shown a correlation between social contact behaviors and the burden of intestinal parasites [65]. Additionally, the presence of humid subtropical climatic conditions of the study area might also be critical, providing essential moisture and optimal temperature for the rapid embryonation of geo‐helminth eggs, like *Ascaris* [66], suggesting the likelihood of parasitic cycling and reinfection.

We first detected the egg of *Controrchis* sp., a dicrocoeliid trematode that dwells in the gallbladder, in a fecal sample from a macaque. However, the prevalence observed is notably lower than that reported to be 89% in black howler monkeys (*Alouatta caraya*) in Belize [42]. Since these flukes rely on a gastropod and an ant as their first and second intermediate hosts, respectively, infection in the macaque may therefore have occurred through the ingestion of ants or other arthropods that consume slime balls excreted by snails, the first intermediate hosts [42, 67].

We also observed a dominant monoparasitism over mixed pattern of infections in consistence with the findings of other studies [27, 68]. However, it was observed that cysts of *Entamoeba* spp., *B. coli*, and oocysts of *Cryptosporidium* sp. were associated with higher co‐infection. Since, these protozoa are pathogenic and can induce either asymptomatic or symptomatic pathologies [59, 69, 70], their co‐infection might be a health‐determining factor in current macaques.

Finally, we compared the parasitic taxa common among humans and macaques in the study area. Notably, only three parasites, *Cryptosporidium* sp., *Taenia* sp., and *A. lumbricoides*, have been reported in humans, and all of these taxa are also prevalent in macaques. Considering the parasitic profile of macaques, the presence of *A. lumbricoides* in anthropogenic habitats might be a common phenomenon [14]. Furthermore, zoonotic *Cryptosporidium* spp., including *C. hominis, C. parvum, C. canis, C. felis, C. muris, C. ubiquitum, C. meleagridis*, and *C. andersoni* [71, 72, 73] and Taeniid worms, such as *Taenia solium* (*Cysticerus*) and *Echinococcus granulosus* (G1 genotype) have been reported in macaques in different geographies [74, 75]. These findings highlighted the possibility that parasite‐infected macaques may transmit the parasites to nearby human populations, and this transmission may lead to endemicity under suitable conditions. Our reports indicated the presence of Taeniid eggs in macaque feces, indicating either this host acts as a primary host for *Taenia* species or as a paratenic host by the accidental ingestion of parasite eggs in contaminated areas, although the chance of macaque‐borne zoonosis of Taeniid is very low. The latter might be true for *E. granulosus*, which may use macaques as secondary or intermediate hosts.

Additionally, several parasites that have been detected only in macaque fecal samples, such as *E*. spp., *E. coli, B. coli, Cyclospora* sp., *Giardia* sp., *Endolimax nana, Fasciola* sp., hookworm, and *Trichuris* sp., also have zoonotic potential [14, 28, 76, 77, 78]. This suggests that cross‐transmission may occur and that increasing interactions between humans and parasite‐infected macaques and their continuous shedding of eggs/cysts of zoonotically significant parasites within the temple periphery and nearby human settlements quickly increase concerns for public health.

We also investigated the risk factors for GIP acquisition and transmission in the local population. Interestingly, we identified that gender and occupation influenced GIPs acquisition and parasitic prevalence. For example, the higher prevalence of parasites in females might be attributed to occupational exposure to soil and contaminated water bodies, as most of them were agricultural laborers (29.6%) and lacked education (40.7%), or had attained only a primary level of education (33.3%). In this scenario, the females working in agricultural farms may be at higher risk to STHs, and in the lack of adequate knowledge concerning parasite and their transmission dynamics, they might not adapt healthy hygienic rules and pay negligence to consuming anti‐parasitic drugs on a periodic basis, which may lead to higher GIP infection. Second, GIPs were highly present among unemployed individuals, housewives, farmers, businessmen, and laborers; however, contrasting results were present among students and teachers, indicating that a lack of education is an essential factor for GIP predominance, although several confounding factors may exist. Notably, the presence of animals, macaque visits, and defecation in human‐inhabitant areas did not significantly affect GIP infections in humans.

The current study has three main limitations. First, it is a cross‐sectional research and, thus, has mainly collected data once. The parasitic diversity and prevalence are dynamic within the same individual or in a population and change with the spatiotemporal context [79, 80]. Second, the purposive sampling size of the current human population is only 100, which has mainly reduced the small sample size within subgroups during risk factor analysis. Therefore, during the analysis of risk factors, there might be problems with Type I and Type II errors that could generate bias toward the application of this study, especially in a more densely populated region. These two limitations can be minimized through further studies with larger population samples, using either longitudinal or controlled designs, to better understand how parasites are distributed within the current population. Third, we observed four morphologically different cysts of *Entamoeba*. Since cyst maturation involves progressive changes in the number of nuclei, relying solely on morphology can be unreliable for differentiating species such as *E. histolytica*, *E. dispar*, and *E. moshkovskii* [81]. Thus, their detection using microscopic examination, or staining methods, or enzyme immunoassays, or rapid immunochromatographic cartridge assay is not perfectly sensitive compared to molecular approaches [82, 83, 84, 85], indicating further PCR and sequence analysis would be helpful to confirm epidemiological patterns of *Entamoeba* species in the study landscapes. Despite these limitations, the findings offer valuable insights that could inform public health strategies and guide integrated human‐animal health programs at both local and regional levels.

## 5 Conclusion

In conclusion, this study revealed greater diversity, prevalence, and concurrency of GIPs in macaques than in humans residing in similar ecological landscapes. Additionally, the detection of morphologically similar forms of *Entamoba* cyst and ova of *Ascaris* and Taeniid, in both hosts, suggests potential cross‐species transmission, though molecular identification is necessary to confirm this. Additionally, increasing human exposure to parasite‐infected macaques involving numerous potentially amphixenotic parasites may exacerbate this situation. While periodic deworming may reduce parasitic infections in humans, effective management of the expanding macaque population is also essential to mitigate zoonotic risks. Furthermore, the One Health concept of research involving the assessment of parasites from human and macaque fecal samples, including nearby soil and water samples, will be crucial for further epidemiological studies that define and evaluate potential sources of amphixenotic risk.

## Author Contributions

**Sabina Chhetala:** conceptualization, field and laboratory survey, methodology; data analysis, original draft preparation, reviewing and editing. **Roshan Babu Adhikari:** conceptualization, laboratory survey, original draft preparation, writing, reviewing, and editing. **Janak Raj Subedi:** conceptualization, reviewing and editing, supervision. **Tirth Raj Ghimire:** conceptualization, data analysis, writing, reviewing and editing, supervision. Sabina Chhetala and Roshan Babu Adhikari contributed equally regarding laboratory analysis and manuscript preparation. All authors approved the final version of the manuscript.

## Disclosure

All authors have read and approved the final version of the manuscript concerning the research, authorship, and/or publication of this article. Two authors (Sabina Chhetala and Roshan Babu Adhikari) contributed equally regarding laboratory analysis and manuscript preparation. The corresponding author had full access to all of the data in this study and takes complete responsibility for the integrity of the data and the accuracy of the data analysis.

## Conflicts of Interest

The authors declare no conflicts of interest.

## Transparency Statement

The lead author Sabina Chhetala affirms that this manuscript is an honest, accurate, and transparent account of the study being reported; that no important aspects of the study have been omitted; and that any discrepancies from the study as planned (and, if relevant, registered) have been explained.

## Supporting information


**Supplementary 1:** Questionnaires and Consent Forms.

**Supplementary 2:** Average (minimum to maximum) size and numbers of specimen images measured. N= Numbers of parasite images, l=length, b=breadth, D= diameter.

**Supplementary 3:** Characteristic features and prevalence of *Entamoeba* spp. Total sample examined (N) = 100.

## Data Availability

The authors confirm that the data supporting the findings of this study are available within the article and in its supporting materials.
