# Supplementary material for: Gastrointestinal Parasites in Humans and Rhesus Macaques: A Cross‐Sectional Study in Bhaktapur, Nepal
Source: Health Sci Rep. 2025 Nov 26;8(12):e71568. doi: 10.1002/hsr2.71568 (PMC12657630; doi:10.1002/hsr2.71568)
Supplement: Supplementary file 1 — Supplementary 1: Questionnaires and Consent Forms. [file HSR2-8-e71568-s001.docx]

**TITLE: Gastrointestinal Parasites in Humans and Rhesus Macaques: A Cross-Sectional Study in Bhaktapur, Nepal**

Sabina Chhetala^1^, Roshan Babu Adhikari ^2,3,4,5^, Janak Raj Subedi^1^, Tirth Raj Ghimire^5,6,*^

^1^Central Department of Zoology, Institute of Science and Technology, Tribhuvan University, Kathmandu, Nepal; Email: [sabinachhetala@gmail.com](mailto:sabinachhetala@gmail.com) (SC), ORCID ID: <https://orcid.org/0009-0001-5693-5863> (SC)

E-mail: [janzoology@gmail.com](mailto:janzoology@gmail.com) (JRS), ORCID ID: <https://orcid.org/0000-0003-2789-9039> (JRS)

^2^Alka Health Institute Pvt. Ltd., Lalitpur, Nepal

^3^Nepalese Army Institute of Health Sciences (NAIHS), Kathmandu, Nepal

^4^Third Pole Conservancy (TPC), Bhaktapur, Nepal

^5^Animal Research Laboratory, Faculty of Science, Nepal Academy of Science and Technology (NAST), Lalitpur, Nepal

E-mail: [srkroshanbabu@gmail.com](mailto:srkroshanbabu@gmail.com) (RBA), ORCID ID: <https://orcid.org/0000-0002-5876-667X> (RBA)

^6^Department of Zoology, Tri-Chandra Multiple Campus, Tribhuvan University, Kathmandu, Nepal

E-mail: [tirth.ghimire@trc.tu.edu.np](mailto:tirth.ghimire@trc.tu.edu.np) (TRG), ORCID ID: <https://orcid.org/0000-0001-9952-1786> (TRG)

***Correspondence:** Dr. Tirth Raj Ghimire, E-mail: [tirth.ghimire@trc.tu.edu.np](mailto:tirth.ghimire@trc.tu.edu.np)

**Supplementary 1: Questionnaires and Consent forms**

प्रश्नावली संख्या …..

**नीलबाराही क्षेत्र भक्तपुरमा रहेको बाँदर र मान्छेको दिशामा पाइने परजीविहरु सम्बन्धि अनुसन्धानको प्रश्नावली**

(“Gastrointestinal parasites in Human and Rhesus Monkey of Nilbarahi area, Bhaktapur, Nepal”)

प्राणीशास्त्र केन्द्रिय विभाग, त्रि.वि.वि कीर्तिपूर र नेपाल विज्ञान तथा प्रविधि प्रज्ञा प्रतिष्ठानद्वारा गरिएको अनुसन्धान

अध्ययन क्षेत्र ………….. टोल……………… जी पी एस…………..

1. अन्तरवार्ता दिनेको लिङ्ग ?

| पुरुष | महिला |
| --- | --- |
| १ | २ |

1. अन्तरवार्ता दिनेको उमेर ?

| १८-४० वर्ष | ४१-६० वर्ष | ६० वर्ष भन्दा माथी |
| --- | --- | --- |
| ३ | ४ | ५ |

1. घर वरीपरी भएका जनावरहरु ?

| कुकूर | बाँदर | कुखुरा | गाई | भैंसी | भेंडा | बाख्रा | सुँगुर | बंगुर |
| --- | --- | --- | --- | --- | --- | --- | --- | --- |
| ६ | ७ | ८ | ९ | १० | ११ | १२ | १३ | १४ |

1. शिक्षाको स्तर कति छ ?

| नपढेको | प्राइमरी सम्म | १० कक्षा सम्म | उच्च शिक्षा (१२ कक्षा- पी. एच. डि.) |
| --- | --- | --- | --- |
| १५ | १६ | १७ | १८ |

1. काम के गर्नुहुन्छ ?

| बेरोजगारी | विद्यार्थी | किसान | ब्यापारी | शिक्षक | मजदुर | सरकारी कर्माचारी |
| --- | --- | --- | --- | --- | --- | --- |
| १९ | २० | २२ | २३ | २४ | २५ | २६ |

1. तपाइँलाई आन्द्राको परजीवी भन्ने थाहा छ ? यदि थाहा छ भने ६ (क) मा जानुस

| छ | छैन |
| --- | --- |
| २७ | २८ |

1. (क) तपाइँलाई कुन-कुन परजीवी आन्द्रामा पाईन्छ भन्ने थाहा छ ?

| गोलो जुका | अंकुशे जुका | फित्त्ते जुका | आउँपर्ने परजीवी | याद छैन | भन्दिन |
| --- | --- | --- | --- | --- | --- |
| २९ | ३० | ३१ | ३२ | ३३ | ३४ |

1. तपाइँलाई मान्छे र कुकुरबीच परजीवी सर्छ भन्ने थाहा छ ?

| छ | छैन |
| --- | --- |
| ३५ | ३६ |

यदि थाहा छ, भने कुन-कुन परजीवी सर्छन् ?

| जिआर्डीया | अंकुशे जुका | गोलो जुका | एन्टअमिबा | अन्य १ | अन्य २ |
| --- | --- | --- | --- | --- | --- |
| ३७ | ३८ | ३९ | ४० | ४१ | ४२ |

1. तपाइँलाई ३ महिना भित्र तलका मध्ये निम्न कुनै रोग वा लक्षण देखा परेको छ ?

| पखाला | कब्जियेत | दिशामा रगत | दिशामा सिंगान रगत | वान्ता | वाकवाकी | पेट दुख्ने | परेको छैन | भन्दिन | थाहा छैन |
| --- | --- | --- | --- | --- | --- | --- | --- | --- | --- |
| ४३ | ४४ | ४५ | ४६ | ४७ | ४८ | ४९ | ५० | ५१ | ५२ |

1. के तपाइँले जुकाको औषधी खानु भाको छ ?

| छ | छैन |
| --- | --- |
| ५३ | ५४ |

छ , भने कहिले खानु भयो ?

| १ महिना भित्र | २-३ महिना भित्र | ४-६ महिना भित्र | ७-१२ महिना भित्र | १२ महिना भन्दा पहिले |
| --- | --- | --- | --- | --- |
| ५५ | ५६ | ५७ | ५८ | ५९ |

1. पेटसम्बन्धि रोगको लक्षण देखा परेमा उपचारको लागी कहाँ जानु हुन्छ ?

| अस्पताल | मेडिकल | धामी झाक्री | घरमै  जडिबुटी | आयुर्बेद औषद्यालय | कतै पनि जाँदिन |
| --- | --- | --- | --- | --- | --- |
| ६० | ६१ | ६२ | ६३ | ६४ | ६५ |

1. घरको वातावरणीय अवस्था कस्तो छ? ( चर्पी सरसफाई र गाइवस्तु वा तिनका दिशाहरु नजिक भए, नभएको आधारमा )

| उत्तम | ठिकै | खराब |
| --- | --- | --- |
| ६६ | ६७ | ६८ |

1. तपाईंको घरमा कस्तो प्रकारको चर्पी प्रयोग गर्नुहुन्छ ?

| खुल्ला दिशा | कच्ची चर्पीको प्रयोग | पक्की चर्पीको प्रयोग |
| --- | --- | --- |
| ६९ | ७० | ७१ |

1. पिउने पानी कहाँ बाट प्रयोग गर्नुहुन्छ ?

| पोखरी | ईनार | कुवा | धारा | जारको पानी |
| --- | --- | --- | --- | --- |
| ७२ | ७३ | ७४ | ७५ | ७६ |

1. खाने पानी कसरी प्रयोग गर्नुहुन्छ ?

| क्लोरिन | उमाल्ने | फिल्टर | उमाल्ने + फिल्टर | सोडिस प्रविधी | उपचार नगरी |
| --- | --- | --- | --- | --- | --- |
| ७७ | ७८ | ७९ | ८० | ८१ | ८२ |

1. कुन जनावरसंग धेरै जसो नजिक बस्नु हुन्छ ?

| कुकूर | बाँदर | कुखुरा | गाई | भैंसी | भेंडा | बाख्रा | बंगुर |
| --- | --- | --- | --- | --- | --- | --- | --- |
| ८३ | ८४ | ८५ | ८६ | ८७ | ८८ | ८९ | ९० |

1. घरमा कुकुर छ ?

| छ | छैन |
| --- | --- |
| ९१ | ९२ |

1. कुकुरलाई जुकाको औषधी खुवाउनु भएको वा इन्जेक्शन लगाउनु भएको छ ?

| छ | छैन |
| --- | --- |
| ९३ | ९४ |

छ , भने कहिले ?

| १ महिना भित्र | १-३ महिना भित्र | ४-६ महिना भित्र | ७-१२ महिना भित्र | १२ महिनाभन्दा पहिले |
| --- | --- | --- | --- | --- |
| ९५ | ९६ | ९७ | ९८ | ९९ |

1. कुकुरलाई दिशा गराउन कहाँ लैजानु हुन्छ ?

| खुल्ला ठाउँमा | खोरमै गर्छन |
| --- | --- |
| १०० | १०१ |

1. बाँदरहरु यो क्षेत्रमा कति आउँछन् ?

| एक महिनामा एउटा | एक महिनामा २ भन्दा बढी |
| --- | --- |
| १०२ | १०३ |

1. बाँदरहरुले वरिपरी दिशा गर्छन ?

| गर्छन | गर्दैनन् |
| --- | --- |
| १०४ | १०५ |

1. बाँदरको दिशा के गर्नुहुन्छ ?

| मल | फालिदिने | केहि पनि नगर्ने | अन्य |
| --- | --- | --- | --- |
| १०६ | १०७ | १०८ | १०९ |

1. बाँदरबाट मान्छेमा पेट सम्बन्धि रोग सर्छ भन्ने थाहा छ ?

| छ | छैन |
| --- | --- |
| ११० | १११ |

छ, भने कुन-कुन रोग सर्छ ?

| पेट दुख्ने | पखाला लाग्ने | वान्ता हुने | अन्य |
| --- | --- | --- | --- |
| ११२ | ११३ | ११४ | ११५ |

नमुना सिरियल………….. मान्छेको रिपोर्ट सङ्कलन गरेको समय र मिती………………….. जी पी एस…………..

| नमुनाको रंग | कालो  ११६ | हरियो  ११७ | रातो  ११८ | सेतो  ११९ | पहेलो  १२० | खैरो  १२१ |
| --- | --- | --- | --- | --- | --- | --- |
| नमुनामा रगत | छ  १२२ | छैन  १२३ |  | हेल्मिन्थ | छ  १२४ | छैन  १२५ |
| आकार प्रकार र रुप | साह्रो  १२६ | गीलो  १२७ | पखाला  १२८ | पानी जस्तै  १२९ |  |  |

नमुना सिरियल………….. कुकुरको रिपोर्ट सङ्कलन गरेको समय र मिती………………….. जी पी एस…………..

| नमुनाको रंग | कालो  १३० | हरियो  १३१ | रातो  १३२ | सेतो  १३३ | पहेलो  १३४ | खैरो  १३५ |
| --- | --- | --- | --- | --- | --- | --- |
| नमुनामा रगत | छ  १३६ | छैन  १३७ |  | हेल्मिन्थ | छ  १३८ | छैन  १३९ |
| आकार प्रकार र रुप | साह्रो  १४० | गीलो  १४१ | पखाला  १४२ | पानी जस्तै  १४३ |  |  |

नमुना सिरियल………….. बाँदरको रिपोर्ट सङ्कलन गरेको समय र मिती………………….. जी पी एस…………..

| नमुनाको रंग | कालो  १४४ | हरियो  १४५ | रातो  १४६ | सेतो  १४७ | पहेलो  १४८ | खैरो  १४९ |
| --- | --- | --- | --- | --- | --- | --- |
| नमुनामा रगत | छ  १५० | छैन  १५१ |  | हेल्मिन्थ | छ  १५२ | छैन  १५३ |
| आकार प्रकार र रुप | साह्रो  १५४ | गीलो  १५५ | पखाला  १५६ | पानी जस्तै  १५७ |  |  |

**cWoog / cg';Gwfgdf efulng rfxg] JolQmsf] cg'dlt**

**g]kfn lj1fg tyf k|ljlw k|1f k|lt:7fgåf/f ug{ nfluPsf] cg';Gwfg .**

**cg';Gwfgsf] ljifo M** gLnaf/fxL If]q eQmk'/df /x]sf] afFb/ / dfG5]sf] lbzfdf kfOg] k/hLljx? ;DalGw cg';Gwfg

(“Gastrointestinal parasites in Human and Rhesus Monkey of Nilbarahi area, Bhaktapur, Nepal”)

pQ/lbg] JolQmnfO{ lgDg s'/fx? /fd|/L a'emfO{Psf] / tnsf] of] ;lx5fkn] ætkfO{+ of] cWoogdf :j]lR5s?kdf pkl:yt e} cfkm'n] hfg]sf] ;To–tYo s'/fx? k|Zg ;f]Wg]nfO{ eg]sf]Æ eGg] hgfpF5 M

- o; cWoogsf] sf/0f / pb]Zosf] af/]df hfgsf/L lbOPsf] .
- o; cWoogdf pQ/ lbg rfxg]x?sf] nflu dfq k|Zg ;f]lwPsf] . pQ/lbP afkt s'g}klg k};f pknAw gu/fOPsf] .
- k|Zg ;f]Wg'eGbf klxnf k|Zg b]vfOPsf] / k|Zgsf] af/]df hfgsf/L lbOPsf] .
- pQ/lbg] JolQmx?sf] ;"rgf uf]Ko /fVg nfluPsf] .
- pQ/lbg] JolQm (!* jif{ eGbf dfyLsf JolQmx?sf] nfuLdfq) lbzfsf] gd"gf jf :ofDkn hDdf ul/Psf] .
- dfG5] / afFb/ a:g] af;:yfgsf] cj:yfsf] t:jL/ lng tyf ltgLx?nfO{ s'g}klg k|sf/sf] c;/ gkg]{ul/ ltgLx?sf] lbzf ;Íng ug{ / t:jL/ lngsf] nflu cg'dtL lnPsf] .
- o; cWoogdf cfPsf] kl/0ffdnfO{ JolQmut klxrfg gv'Ng] u/]/ ljleGg dfWod -hg{n, k':ts, cflb_ df k|sflzt ul/g]5 .

cGt/jft{f lng]sf] gfd M cGt/jft{f lbg]sf] gfd M

;xL5fk ;xL5fk

ldltM ldltM
