# Supplementary material for: Gastrointestinal Parasites in Humans and Rhesus Macaques: A Cross‐Sectional Study in Bhaktapur, Nepal
Source: Health Sci Rep. 2025 Nov 26;8(12):e71568. doi: 10.1002/hsr2.71568 (PMC12657630; doi:10.1002/hsr2.71568)
Supplement: Supplementary file 2 — Supplementary 2: Average (minimum to maximum) size and numbers of specimen images measured. N= Numbers of parasite images, l=length, b=breadth, D= diameter. [file HSR2-8-e71568-s003.docx]

**TITLE: Gastrointestinal Parasites in Humans and Rhesus Macaques: A Cross-Sectional Study in Bhaktapur, Nepal**

Sabina Chhetala^1^, Roshan Babu Adhikari ^2,3,4,5^, Janak Raj Subedi^1^, Tirth Raj Ghimire^5,6,*^

^1^Central Department of Zoology, Institute of Science and Technology, Tribhuvan University, Kathmandu, Nepal; Email: [sabinachhetala@gmail.com](mailto:sabinachhetala@gmail.com) (SC), ORCID ID: <https://orcid.org/0009-0001-5693-5863> (SC)

E-mail: [janzoology@gmail.com](mailto:janzoology@gmail.com) (JRS), ORCID ID: <https://orcid.org/0000-0003-2789-9039> (JRS)

^2^Alka Health Institute Pvt. Ltd., Lalitpur, Nepal

^3^Nepalese Army Institute of Health Sciences (NAIHS), Kathmandu, Nepal

^4^Third Pole Conservancy (TPC), Bhaktapur, Nepal

^5^Animal Research Laboratory, Faculty of Science, Nepal Academy of Science and Technology (NAST), Lalitpur, Nepal

E-mail: [srkroshanbabu@gmail.com](mailto:srkroshanbabu@gmail.com) (RBA), ORCID ID: <https://orcid.org/0000-0002-5876-667X> (RBA)

^6^Department of Zoology, Tri-Chandra Multiple Campus, Tribhuvan University, Kathmandu, Nepal

E-mail: [tirth.ghimire@trc.tu.edu.np](mailto:tirth.ghimire@trc.tu.edu.np) (TRG), ORCID ID: <https://orcid.org/0000-0001-9952-1786> (TRG)

***Correspondence:** Dr. Tirth Raj Ghimire, E-mail: [tirth.ghimire@trc.tu.edu.np](mailto:tirth.ghimire@trc.tu.edu.np)

**Supplementary 2:** Average (minimum to maximum) size and numbers of specimen images measured. N= Numbers of parasite images, l=length, b=breadth, D= diameter.

| **GIP species** | **Humans** | | **Macaques** | | **Conclusive notes** |
| --- | --- | --- | --- | --- | --- |
|  | **Dimensions** | **Characters** | **Dimensions** | **Characters** |  |
| ***Cryptosporidium* sp.** | N= 50, D=4 µm (3-7) | Oval, round | N=50, D=4 µm (3-6) | Oval, round | Morphologically similar forms |
| **Taeniid** | N=20, l= 27µm (26-28) x b=26µm (24-28) | Round, oval hooks | N=20, l= 27µm (26-28) x b=22 µm (20-24) | Round, oval hooks | Morphologically similar and slightly smaller forms in macaques |
| **Ascarid** | N=40, l= 32 µm (30-34) x 27 µm (26-28) | Round, ovoidal, thick shell, either corticated or non-corticated, Human *Ascaris*-like eggs | N=40, l= 20 µm (18-22) x b=18 µm (16-20) | i. Round, ovoidal, thick shell, corticated or non-corticated, Human *Ascaris*-like eggs ii. *Toxocara*-like eggs (Ovoidal, thin corticated shell) | Morphologically similar and different forms |
