# Supplementary material for: Gastrointestinal Parasites in Humans and Rhesus Macaques: A Cross‐Sectional Study in Bhaktapur, Nepal
Source: Health Sci Rep. 2025 Nov 26;8(12):e71568. doi: 10.1002/hsr2.71568 (PMC12657630; doi:10.1002/hsr2.71568)
Supplement: Supplementary file 3 — Supplementary 3: Characteristic features and prevalence of Entamoeba spp. Total sample examined (N) = 100. [file HSR2-8-e71568-s002.docx]

**TITLE: Gastrointestinal Parasites in Humans and Rhesus Macaques: A Cross-Sectional Study in Bhaktapur, Nepal**

Sabina Chhetala^1^, Roshan Babu Adhikari ^2,3,4,5^, Janak Raj Subedi^1^, Tirth Raj Ghimire^5,6,*^

^1^Central Department of Zoology, Institute of Science and Technology, Tribhuvan University, Kathmandu, Nepal; Email: [sabinachhetala@gmail.com](mailto:sabinachhetala@gmail.com) (SC), ORCID ID: <https://orcid.org/0009-0001-5693-5863> (SC)

E-mail: [janzoology@gmail.com](mailto:janzoology@gmail.com) (JRS), ORCID ID: <https://orcid.org/0000-0003-2789-9039> (JRS)

^2^Alka Health Institute Pvt. Ltd., Lalitpur, Nepal

^3^Nepalese Army Institute of Health Sciences (NAIHS), Kathmandu, Nepal

^4^Third Pole Conservancy (TPC), Bhaktapur, Nepal

^5^Animal Research Laboratory, Faculty of Science, Nepal Academy of Science and Technology (NAST), Lalitpur, Nepal

E-mail: [srkroshanbabu@gmail.com](mailto:srkroshanbabu@gmail.com) (RBA), ORCID ID: <https://orcid.org/0000-0002-5876-667X> (RBA)

^6^Department of Zoology, Tri-Chandra Multiple Campus, Tribhuvan University, Kathmandu, Nepal

E-mail: [tirth.ghimire@trc.tu.edu.np](mailto:tirth.ghimire@trc.tu.edu.np) (TRG), ORCID ID: <https://orcid.org/0000-0001-9952-1786> (TRG)

***Correspondence:** Dr. Tirth Raj Ghimire, E-mail: [tirth.ghimire@trc.tu.edu.np](mailto:tirth.ghimire@trc.tu.edu.np)

**Supplementary 3:** Characteristic features and prevalence of *Entamoeba* spp. Total sample examined (N) = 100.

| ***Entamoeba* Morphometry** | **Characteristics of cysts** | **No. of cysts/photos measured** | **Length x Breadth (Average) 40x** | **Overall Prevalence (nX100/N)** | **Stool characteristics** |
| --- | --- | --- | --- | --- | --- |
| **A** | Round, Oval, moderate, 4 nuclei, yellow | 500 | 14.5 µm x 13.5 µm | 41% | Liquid, semisolid, solid, red, yellow, green |
| **B** | Round, large, 8 nuclei, | 400 | 17 µm x 17 µm | 37% | Liquid, semisolid, solid |
| **C** | Oval, round, smallest, 4 nuclei | 200 | 4.5 µm x 4.5 µm | 14% | Liquid, semisolid, solid |
| **D** | Oval, round, moderate, 1 nucleus | 100 | 10 µm x 9 µm | 2% | Liquid, semisolid, solid |
